# Sarcoma of the Breast: Clinical Characteristics and Outcomes of 991 Patients from the National Cancer Database

**DOI:** 10.1155/2021/8828158

**Published:** 2021-01-21

**Authors:** Jin Sun Lee, Kevin Yoon, Mykola Onyshchenko

**Affiliations:** ^1^Division of Hematology-Oncology, Harbor-UCLA Medical Center, Torrance, CA, USA; ^2^Department of Medicine, Harbor-UCLA Medical Center, Torrance, CA, USA; ^3^The Lundquist Institute, Torrance, CA, USA

## Abstract

**Background:**

Sarcoma of the breast is a rare malignancy with heterogeneous histology. Angiosarcoma, including secondary angiosarcoma from previous radiation, is the most common type of sarcoma of the breast. Other types of sarcomas of the breast have limited clinical and survival information.

**Methods:**

We obtained clinicopathological data and survival outcomes from the patients with sarcoma of the breast, excluding angiosarcoma, that were registered in the National Cancer Database (NCDB) from 2004 to 2016. The treatment patterns and prognostic factors were analyzed.

**Results:**

A total of 991 patients had sarcoma of the breast other than angiosarcoma. The most common histology was spindle cell sarcoma (13.4%), followed by leiomyosarcoma (11.7%) and giant cell sarcoma (10.1%). Surgical resection was performed in 894 out of 991 patients (90.2%), including R0 resection achieved in 781 (87.4%). The patients who received surgery showed better survival than the patients without surgery regardless of radiation therapy. When radiation was added to the surgical management, the OS (overall survival) benefit was marginally significant (hazard ratio 1.30 (CI 1.01–1.67), *p*=0.044). Adding chemotherapy did not improve OS.

**Conclusions:**

Surgical resection seems to be the most important treatment modality in sarcoma of the breast from the analysis of a large database. Radiation therapy added a minor survival benefit to the patients who received surgical resection. Systemic chemotherapy did not play a clear role in sarcoma of the breast.

## 1. Introduction

Sarcoma of the breast is a rare and diverse group of malignancies that derive from mesenchymal tissues. There are approximately 4.6 new cases per million women per year and account for less than 1% of all breast malignancies [1, 2]. As with other soft tissue sarcomas, the primary sarcoma of the breast is associated with genetic conditions such as Li-Fraumeni syndrome, familial adenomatous polyposis, and neurofibromatosis type 1 [3–5]. They are also associated with certain environmental risk factors including arsenic compounds, vinyl chloride, and alkylators [5]. Secondary sarcoma of the breast most often occurs after radiation therapy for the breast or other intrathoracic malignancies such as nonHodgkin lymphoma [6]. Overall, the most common subtype of sarcoma of the breast is secondary angiosarcoma [7]. Angiosarcoma of the breast has been reported that it is associated with poor prognosis, and mastectomy is the mainstay of the treatment [8, 9]. However, other types of sarcoma of the breast have not been studied mainly due to the rarity and histological heterogeneity of the disease.

Staging of sarcoma of the breast is usually performed using the American Joint Committee on Cancer (AJCC) system for soft tissue sarcomas. Tumor size >5 cm, high-grade disease, angiosarcoma histology, and positive resection margins are associated with worse prognosis [2, 10, 11]. A multidisciplinary approach involving surgeons, radiation specialist, and oncologist is vital for the treatment of sarcomas of the breast, although there is no clear consensus in the treatment regimen.

Complete surgical resection with negative margins remains the mainstay of the treatment. The median 5-year overall survival (OS) is 63.5% based on a major series of sarcoma of the breast [12]. Sarcoma of the breast relatively had a worse prognosis than breast carcinoma [13].

Herein, we demonstrate clinicopathological characteristics of the sarcoma of the breast that is not angiosarcoma in histology. The information regarding 991 patients with sarcoma of the breast from 2004 to 2016 was collected and analyzed from the National Cancer Database (NCDB). This study aims to further elucidate clinical features and their relevance to the survival of the rare sarcoma of the breast.

## 2. Methods

### 2.1. Data Source

The NCDB is one of the largest oncology data collected from more than 1,500 Commission on Cancer- (CoC-) accredited hospitals. About 70% of all newly diagnosed cancer in the United States is captured and reported to the NCDB. This includes patient demographics, initial staging, tumor histology, treatment course, and survival outcomes. The patients who were diagnosed with breast cancer from 2004 to 2016 were collected from the NCDB. Among them, the patients with sarcoma were identified by ICD-O histology codes that start with 88 or 89. Angiosarcoma (ICD-O 9120) was excluded. The following variables were captured: age, gender, race, treatment facilities, tumor histology, tumor grade, TNM stage, the treatment history of surgery, radiation, chemotherapy, duration of follow-up, and survival status.

### 2.2. Statistical Analysis

Survival outcome was obtained by the Kaplan–Meier curve and log-rank tests. The univariate and multivariate Cox proportional hazard model was used to evaluate prognostic indicators. Open statistical software RStudio® was utilized for all the statistical analyses. The graphics for Kaplan–Meier curves were obtained using Jamovi® version 1.2.17.

## 3. Results

### 3.1. Patient Characteristics

A total of 2,696,734 patients were registered with a diagnosis of breast cancer between 2004 and 2016. Among them, 536 patients had angiosarcoma, and 991 patients (0.04%) had histology of sarcoma other than angiosarcoma. The clinical characteristics of these patients are described in Table 1. More than half of the patients were the age of between 51 and 75, and 30% of patients were the age of 50 or younger. Male patients were 3.8% of all the patients, which is higher than the proportion of male patients in breast cancer where generally 1% is male patients.

### 3.2. Tumor Characteristics

Sarcoma of the breast was composed of a variety of histology. A large number of cases (25.4%) were reported as sarcoma, NOS without subtypes of histology. The most common histology of sarcoma was spindle cell sarcoma (13.4%), followed by leiomyosarcoma (11.7%), giant cell sarcoma (10.1%), and stromal sarcoma (6.2%). More than half of sarcoma of the breast showed poorly differentiated or undifferentiated grade. T1 (primary tumor size less than 5 cm) was 45.5% and T2 was 21.5% of the patients per AJCC soft tissue sarcoma staging. Lymph node dissection was performed in 35% of the patients, 33.4% of patients who were treated in academic cancer centers and 38.0% of patients from community programs, and lymph node metastasis (N1) occurred in about 6% of the patients who underwent lymph node dissection. Distant metastasis (M1) was rare.

### 3.3. Treatment

Treatment history is summarized in Table 2. The majority of patients received surgical resection (90.2%), and R0 resection was achieved in 781 out of 894 patients who received surgery (87.4%). Among 894 patients, 269 patients received radiation therapy and 149 patients received chemotherapy (Tables 3 and 4). The patients who did not receive surgery were treated with radiation in 18 out of 97 patients or chemotherapy in 36 out of 97 patients. Regardless of the T stage, most of the patients were attempted for surgical management (88–95% by T stages) as shown in Table 5. More patients with T3-4 received chemotherapy compared with T1-2.

### 3.4. Survival

**Figure 1:**
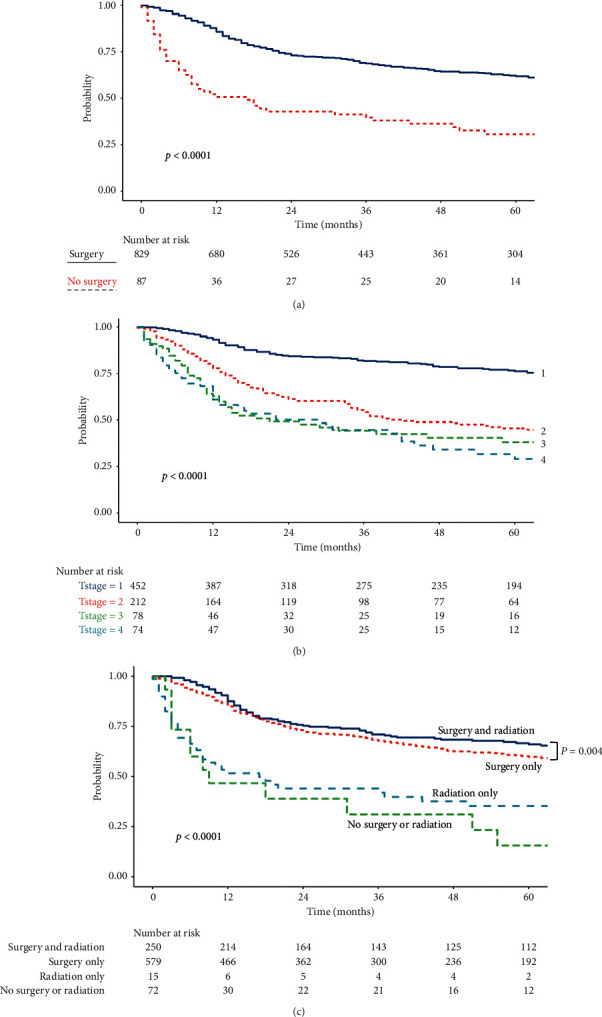
Kaplan–Meyer curve of overall survival. (a) Kaplan–Meyer curve of overall survival by surgical treatment. (b) Kaplan–Meyer curve of overall survival by T stages. (c) Kaplan–Meyer curve of overall survival by surgical and radiation treatment.

The median follow-up time from the date of diagnosis to the date of death or last contact was 36.7 months. Univariate Cox regression was applied to evaluate clinicopathologic factors that affect survival (Table 6). Patient age and treatment facilities affect OS. Patients with the age of 50 or less demonstrated better survival than age over 50. When patients were treated at an academic or research center, they had superior survival. Several tumor characteristics were also related to OS. The factors that carried out good prognosis are well-differentiated tumor grade, early T stage, no LN involvement, no distant metastasis, surgical treatment, and radiation treatment. Interestingly, receiving chemotherapy was associated with poor survival, which may be explanted by the fact that chemotherapy tended to be applied for advanced disease, including the higher T stage. OS based on T stage and treatment modalities is described in Figures 1(a) and 1(b) as a Kaplan–Meyer curve. Patients who received surgery showed better survival compared with patients without surgery regardless of applying radiation. When radiation was added to surgical management, the OS benefit was marginally significant (HR 1.30 (CI 1.01–1.67), *p*=0.044) as shown in Figure 1(c).

## 4. Discussion

The data regarding the optimal management of sarcoma of the breast is limited due to the rarity of the disease. While angiosarcoma is the most common type of sarcoma of the breast that is related to previous radiation and poor prognosis [14, 15], the clinical and therapeutic knowledge of other types of sarcoma of the breast is largely unknown. Complete surgical excision remains the mainstay of treatment in achieving local control and the only potentially curative therapy for sarcoma of the breasts. In our research, surgical excision was associated with superior survival. Historically, total mastectomy had been considered as the gold standard in surgical therapy for sarcoma of the breasts [16]. Numerous studies subsequently demonstrated that wide excision to negative margins provides comparable results [17–19]. Sarcoma of the breasts often present with greater than 5 cm; hence, mastectomy may be the most effective way to achieve the goal of a wide margin [20]. Because soft tissue sarcomas typically spread via hematogenous route, axillary lymph node dissection is discouraged [20]. There are certain subtypes of sarcoma with potential for lymphogenous spread, particularly angiosarcoma, rhabdomyosarcoma, clear cell sarcoma, synovial sarcoma, and epithelioid sarcoma [21]. If nodal metastases are suspected by imaging, a biopsy should be obtained because reactive lymphadenopathy may be seen in up to 25% of sarcoma of the breast patients [22].

In contrast to surgical resection, there is no definitive consensus in using radiation therapy for sarcoma of the breast. However, it may be beneficial in disease with high-risk features. Barrow et al. reviewed 59 patients (16 patients had a segmental resection and 38 had a mastectomy) over 43 years with primary sarcoma of the breast [11]. 4 of 16 patients received radiation after segmental resection and 13 of 38 received radiation after mastectomy. Although there were no statistically significant differences, authors found that 14% of local failure occurred in patients who had segmental resection alone versus segmental resection followed by radiation, and 34% versus 13% local failure was observed in patients who underwent mastectomy compared to patients who had a mastectomy followed by radiation. The authors concluded that radiation therapy is likely beneficial in larger tumor size and positive margins. In another study, Johnstone et al. observed that 10 patients with high-grade sarcoma who underwent a mastectomy and postoperative radiation had a median follow-up for 99 months, thus concluding that high-grade sarcoma of the breasts should be considered for radiation therapy to achieve optimal local control [23]. Our current research demonstrated the marginal benefit of radiation treatment after surgery (*p*=0.044).

The role of chemotherapy for sarcoma of the breast is also uncertain. Gutman et al. demonstrated evidence of increased disease-free survival and improved overall survival for patients who received adjuvant chemotherapy [18]. However, it is difficult to draw any conclusion given that there are no prospective trials specifically assessing the benefit of adjuvant chemotherapy. Most studies that investigated chemotherapy for sarcoma of the breast, especially neoadjuvant chemotherapy was limited, and definitive conclusion could not be drawn [17, 24, 25]. Similar to other sarcomas, doxorubicin and ifosfamide-based regimens can be used [26].

It appears that different types of facilities may affect survival outcomes in cancer treatment. For example, patients treated at NCI (National Cancer Institute) designated cancer centers or academic teaching hospitals have been reported to have superior survival [27]. Our research demonstrated that an academic or research program is related to better OS in sarcoma of the breast.

This study was one of the largest analyses of sarcoma of the breasts up to date. Characteristics and treatment modalities of 991 sarcoma of the breast patients were examined after excluding angiosarcoma that is a largely separate entity of sarcoma of the breast. Predictors for worse survival were consistent with previous studies, which include older age, advanced grade, and stage of the tumor. In addition, treatment facilities were an independent factor associated with OS. This finding may indicate that any patient with sarcoma of the breast should be at least discussed in a multidisciplinary meeting and better operated on in a dedicated center. The majority of patients received R0 resection, and the vast majority of patients did not receive radiation or chemotherapy. When radiation was administered to the patients, however, it was associated with a better outcome. On the other hand, chemotherapy was associated with a worse outcome. This may be due to patients with worse prognoses such as advanced T stages were likely to receive chemotherapy. Our study confirms that surgery is the most commonly accepted treatment for sarcoma of the breast. At the same time, the utilization of radiation and chemotherapy is less uniform.

As with other NCDB-based studies, the study is limited by retrospective analysis from a nationwide database. Not all patients nationwide were reported, and more importantly, only limited information were collected, which did not include the indication to withhold surgery. One could only assume that the patients were not operated due to metastases or irresectable tumors. The date of local recurrence was not reported, so prognostic factors only for OS were analyzed. Other significant details such as complications, prior cancer, or radiation history were not included. Angiosarcoma was excluded from the analysis using ICD-O histology code, but it might be included in some cases that were reported as sarcoma, NOS, whose histology was unable to be confirmed as an individual case.

The analysis of a large number of patients with sarcoma of the breast from the NCDB demonstrates that surgery was the cornerstone of treatment. The findings support the general findings in sarcomas with surgical resection being the mainstay of the treatment and no true gain from any adjuvant treatment. Furthermore, our study would emphasize the importance of dedicated centers for the treatment of sarcoma.

## 5. Conclusion

In this large NCDB study, surgical resection was the critical treatment modality in sarcoma of the breast. Adjuvant treatment did not play a clear role in sarcoma of the breast. Multidisciplinary approach in a dedicated center should be considered for a better clinical outcome.

## Figures and Tables

**Table 1:** Patient and tumor characteristics.

Patient characteristics	Numbers (%)
Age	
≤50	294 (29.7%)
51–75	515 (51.9%)
75<	182 (18.4%)

Gender	
Female	953 (96.2%)
Male	38 (3.8%)

Race	
NonHispanic white	645 (65.1%)
Black	172 (17.4%)
Hispanic	61 (6.2%)
Asian	47 (4.7%)
Others/unknown	66 (6.7%)

Treatment facility	
Academic/research program	327 (33.0%)
Community program	545 (55.0%)
Unknown	119 (12.0%)

Histology	
Sarcoma, NOS	252 (25.4%)
Spindle cell sarcoma	133 (13.4%)
Leiomyosarcoma	116 (11.7%)
Giant cell sarcoma	100 (10.1%)
Stromal sarcoma, NOS	61 (6.2%)
Malignant fibrous histiocytoma	59 (6.0%)
Fibromyxosarcoma	36 (3.6%)
Dermatofibrosarcoma	34 (3.4%)
Fibrosarcoma	34 (3.4%)
Undifferentiated sarcoma	31 (3.1%)
Liposarcoma	31 (3.1%)
Pleomorphic liposarcoma	22 (2.2%)
Others	82 (8.3%)

Tumor grade	
Well differentiated	101 (10.2%)
Moderately differentiated	126 (12.7%)
Poorly differentiated	292 (29.5%)
Undifferentiated	205 (20.7%)
Unknown	267 (26.9%)

T stage	
1	451 (45.5%)
2	213 (21.5%)
3	78 (7.9%)
4	74 (7.5%)
Microinvasion	1 (0.1%)
Unknown	174 (17.6%)

N stage	
0	331^*∗*^
1	22^*∗*^
Unknown	638^†^

M stage	
0	785
1	73
Unknown	133

NOS, not otherwise specified. ^*∗*^Lymph nodes were surgically removed and examined. ^†^Lymph node sampling/dissection was not performed.

**Table 2:** Summary of treatment: treatment modality.

Treatment modality	Numbers (%)
Surgery	
Yes	894 (90.2%)
No	97 (9.8%)
Radiation therapy	
Yes	287 (29.0%)
No	704 (71.0%)
Chemotherapy	
Yes	185 (18.7%)
No	806 (81.3%)

**Table 3:** Summary of treatment: multimodality treatment with radiation.

	Radiation therapy	No radiation therapy
Surgery	269	625
No surgery	18	79

**Table 4:** Summary of treatment: multimodality treatment with chemotherapy.

	Chemotherapy	No chemotherapy
Surgery	149	745
No surgery	36	61

**Table 5:** Summary of treatment: treatment by T stage.

T stage	Surgery		Radiation		Chemotherapy	
	Yes (%)	No (%)	Yes (%)	No (%)	Yes (%)	No (%)
1	428 (94.7%)	24 (5.3%)	117 (25.9%)	335 (74.1%)	45 (10.0%)	407 (90.0%)

2	190 (89.2%)	23 (10.8%)	81 (38.0%)	132 (62.0%)	54 (25.4%)	159 (74.6%)

3	69 (88.5%)	9 (11.5%)	34 (43.6%)	44 (56.4%)	25 (32.1%)	53 (67.9%)

4	66 (98.2%)	8 (10.8%)	20 (27.0%)	54 (73.0%)	25 (33.8%)	49 (66.2%)

Unknown	141	33	35	139	36	138

Total	894	97	287	704	185	806

**Table 6:** Overall survival associated with clinicopathologic characteristics, the Cox proportional hazard model.

Characteristics	Number	HR	95% CI	*p*
Age				
≤50	275	1	NA	NA
51–75	472	1.45	1.10–1.92	0.008
75<	169	4.41	3.30–5.93	<0.001

Treatment facility				
Academic/research	294	1	NA	NA
Community hospital	510	1.30	1.03–1.63	0.024

Tumor grade				
Well differentiated	96	1	NA	NA
Moderately differentiated	110	1.60	0.91–2.82	0.101
Poorly differentiated/undifferentiated	459	2.99	1.87–4.87	<0.001

T stage				
T1	452	1	NA	NA
T2	212	2.52	1.93–3.28	<0.001
T3	78	3.61	2.54–5.12	<0.001
T4	74	3.99	2.81–5.64	<0.001

N stage				
N0	310	1	NA	NA
N1	21	3.34	1.91–5.83	<0.001

M stage				
M0	785	1	NA	NA
M1	73	6.51	4.90–8.65	<0.001

Surgery				
Yes	829	1	NA	NA
No	87	2.87	2.13–3.85	<0.001

Surgical margins				
Negative (R0)	720	1	NA	NA
Positive	70	2.40	1.73–3.33	<0.001

Radiation				
Yes	265	1	NA	NA
No	651	1.28	1.01–1.62	0.038

Chemotherapy				
Yes	171	1	NA	NA
No	745	0.69	0.54–0.88	0.003

NA, not applicable.

## Data Availability

The data that support the findings of this study are available from the National Cancer Database. Restrictions may apply to the availability of these data, which were used under permit-required access for this study.

## References

[1] McGowan T. S., Cummings B. J., O’Sullivan B., Catton C. N., Miller N., Panzarella T. (2000). An analysis of 78 sarcoma of the breast patients without distant metastases at presentation. *International Journal of Radiation Oncology, Biology, Physics*.

[2] Surov A., Holzhausen H.-J., Ruschke K. (2011). Primary and secondary breast lymphoma: prevalence, clinical signs and radiological features. *Acta Radiologica*.

[3] Birch J. M., Alston R. D., McNally R. J. (2001). Relative frequency and morphology of cancers in carriers of germline TP53 mutations. *Oncogene*.

[4] Malkin D., Li F. P., Strong L. C. (1990). Germ line p53 mutations in a familial syndrome of breast cancer, sarcomas, and other neoplasms. *Science*.

[5] Lahat G., Lazar A., Lev D. (2008). Sarcoma epidemiology and etiology: potential environmental and genetic factors. *Surgical Clinics of North America*.

[6] Penel N., Grosjean J., Robin Y. M., Vanseymortier L., Clisant S., Adenis A. (2008). Frequency of certain established risk factors in soft tissue sarcomas in adults: a prospective descriptive study of 658 cases. *Sarcoma*.

[7] Guibout C., Adjadj E., Rubino C. (2005). Malignant breast tumors after radiotherapy for a first cancer during childhood. *Journal of Clinical Oncology*.

[8] Scow J. S., Reynolds C. A., Degnim A. C., Petersen I. A., Jakub J. W., Boughey J. C. (2010). Primary and secondary angiosarcoma of the breast: the Mayo clinic experience. *Journal of Surgical Oncology*.

[9] Arora T. K., Terracina K. P., Soong J., Idowu M. O., Takabe K. (2014). Primary and secondary angiosarcoma of the breast. *Gland Surgery*.

[10] Barnes L., Pietruszka M. (1977). Sarcomas of the breast. A clinicopathologic analysis of ten cases. *Cancer*.

[11] Barrow B. J., Janjan N. A., Gutman H. (1999). Role of radiotherapy in sarcoma of the breast—a retrospective review of the M. D. Anderson experience. *Radiotherapy and Oncology*.

[12] Adem C., Reynolds C., Ingle J. N., Nascimento A. G. (2004). Primary breast sarcoma: clinicopathologic series from the Mayo clinic and review of the literature. *British Journal of Cancer*.

[13] Lim S. Z., Ong K. W., Tan B. K. T., Selvarajan S., Tan P. H. (2016). Sarcoma of the breast: an update on a rare entity. *Journal of Clinical Pathology*.

[14] Kunkiel M., Maczkiewicz M., Jagiełło-Gruszfeld A., Nowecki Z. (2018). Primary angiosarcoma of the breast-series of 11 consecutive cases-a single-centre experience. *Current Oncology*.

[15] Yin M., Wang W., Drabick J. J., Harold H. A. (2017). Prognosis and treatment of non-metastatic primary and secondary breast angiosarcoma: a comparative study. *BMC Cancer*.

[16] Berg J. W., Decrosse J. J., Fracchia A. A., Farrow J. (1962). Stromal sarcomas of the breast. A unified approach to connective tissue sarcomas other than cystosarcoma phyllodes. *Cancer*.

[17] Bousquet G., Confavreux C., Magné N. (2007). Outcome and prognostic factors in breast sarcoma: a multicenter study from the rare cancer network. *Radiotherapy and Oncology*.

[18] Gutman H., Pollock R. E., Ross M. I. (1994). Sarcoma of the breast: implications for extent of therapy. The M. D. Anderson experience. *Surgery*.

[19] Zelek L., Llombart-Cussac A., Terrier P. (2003). Prognostic factors in primary breast sarcomas: a series of patients with long-term follow-up. *Journal of Clinical Oncology*.

[20] Hsu C., Mccloskey S. A., Peddi P. F. (2016). Management of breast sarcoma. *Surgical Clinics of North America*.

[21] Sherman K. L., Kinnier C. V., Farina D. A. (2014). Examination of national lymph node evaluation practices for adult extremity soft tissue sarcoma. *Journal of Surgical Oncology*.

[22] Christensen L., Schiødt T., Blichert-Toft M., Hansen J. P., Hansen O. H. (1988). Sarcomas of the breast: a clinico-pathological study of 67 patients with long term follow-up. *European Journal of Surgical Oncology*.

[23] Johnstone P. A. S., Pierce L. J., Merino M. J., Yang J. C., Epstein A. H., DeLaney T. F. (1993). Primary soft tissue sarcomas of the breast: local-regional control with post-operative radiotherapy. *International Journal of Radiation Oncology, Biology, Physics*.

[24] Fields R. C., Aft R. L., Gillanders W. E., Eberlein T. J., Margenthaler J. A. (2008). Treatment and outcomes of patients with primary breast sarcoma. *The American Journal of Surgery*.

[25] McGregor G. I., Knowling M. A., Este F. A. (1994). Sarcoma and cystosarcoma phyllodes tumors of the breast—a retrospective review of 58 cases. *The American Journal of Surgery*.

[26] Patel S. R., Vadhan-Raj S., Burgess M. A. (1998). Results of two consecutive trials of dose-intensive chemotherapy with doxorubicin and ifosfamide in patients with sarcomas. *American Journal of Clinical Oncology*.

[27] Pfister D. G., Rubin D. M., Elkin E. B. (2015). Risk adjusting survival outcomes in hospitals that treat patients with cancer without information on cancer stage. *JAMA Oncology*.

